# Significance of examined lymph nodes number and metastatic lymph nodes ratio in overall survival and adjuvant treatment decision in resected laryngeal carcinoma

**DOI:** 10.1002/cam4.2902

**Published:** 2020-02-29

**Authors:** Xiaoke Zhu, Min Zhao, Liang Zhou, Ming Zhang, Pengyu Cao, Lei Tao

**Affiliations:** ^1^ Department of Otolaryngology Shanghai Key Clinical Disciplines of otorhinolaryngology Eye Ear Nose and Throat Hospital Fudan University Shanghai China; ^2^ School of Nursing Fudan University Shanghai China

**Keywords:** cancer risk factors, head and neck cancer, nomogram, survival

## Abstract

**Objective:**

The value of adjuvant therapy in resected laryngeal cancer remains controversial. This large SEER‐based cohort study aimed to investigate the existing parameters of lymph node status that could predict survival outcomes and the prognostic value of adjuvant treatment in resected laryngeal carcinoma.

**Methods:**

Population‐based data from the US Surveillance, Epidemiology, and End Results (SEER‐18) Program on patients after laryngectomy and lymphadenectomy (2004‐2015) were analyzed. The optimal cut‐off values for examined lymph nodes number (ELNs) and metastatic lymph nodes ratio (MLNR) were determined using the X‐tile program. Associations of ELNs and MLNR with overall survival were investigated through Cox regression analysis. A survival‐predicting model was then constructed to stratified patients. The prognostic value of adjuvant therapy was evaluated in different subgroups.

**Results:**

A total of 2122 patients with resected laryngeal cancer were analyzed. A novel survival‐predicting model was proposed based on ELNs, MLNR, and other clinicopathological characteristics. Patients were stratified into three subgroups with the increasing risk of death. Only patients in the high‐risk group who receiving adjuvant treatment had a significantly better survival outcome than those receiving surgery alone.

**Conclusion:**

A new survival‐predicting model was established in this study, which was superior in assessing the survival outcomes of patients with resected laryngeal cancer. Notably, this model was also able to assist in the decision making of adjuvant therapy for patients and physicians.

## Graphical abstract

In this study, we jointly use of ELNs, MLNR, age and T stage to construct a novel survival‐predicting model, which was superior in evaluating the survival outcomes of patients with resected laryngeal cancer. Notably, this model was also able to stratified patients and evaluate the prognostic value of adjuvant therapy. Adjuvant therapy was recommended for patients in high‐risk group.

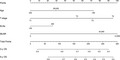

## 1 INTRODUCTION

Laryngeal carcinoma (LC) ranks second among the most common cancers in the head and neck region, with an estimated incidence of 82 000 deaths and 151 000 new cases annually around the world.^1^ According to its anatomic subsites, laryngeal cancer is generally divided into glottic, supraglottic, and subglottic carcinoma with squamous cell cancer as the vast majority of histological type.^2^ With public awareness of excessive alcohol consumption and smoking as major predisposing factors, the incidence of laryngeal carcinoma has decreased over the past decade.^3^ However, according to a retrospective study from the US National Cancer Data Base (NCDB), the survival outcomes of laryngeal cancer patients have unexpectedly declined, whereas that of the most other tumor locations have improved.^4^

Surgical resection remains an integral part of the treatment of LC with the emphasis on maximal oncologic control and optimal functional outcomes, including partial or total laryngectomy.^5^ In addition to surgery, adjuvant therapy is reckoned as an essential supplementary therapy for subclinical foci elimination, however, inappropriate adjuvant therapy may induce adverse effects for patients, including tissue necrosis, laryngeal edema xerostomia, trismus, and fibrosis.^6^ To date, the values of adjuvant therapies, including radiotherapy, chemotherapy, and chemoradiotherapy, remain controversial, partially due to the failure to select suitable LC patients.

Several previous studies have demonstrated the pN stage as a prognostic predictor and an efficient indicator for adjuvant therapy, which is mainly based on the number of metastatic lymph nodes and extra‐nodal extension (ENE).^7^, ^8^ However, a sufficient number of the examined lymph node is key to ensure the precise nodal staging. The number of examined lymph nodes (ELNs) can reflect the extent of lymphadenectomy and has been reported to be associated with oncological outcomes in several cancers.^9^, ^10^ Some studies also reported that the ratio of metastatic lymph nodes (MLNR) is a prognostic factor in certain tumors, including esophageal carcinoma and medullary thyroid cancer.^11^, ^12^

In this study, we aimed to investigate the prognostic value of ELNs and MLNR for patients with laryngeal cancer who had undergone laryngectomy and lymphadenectomy. This study also explored the predictive value of the combination of ELNs, MLNR, and other clinicopathological features in the decision making of adjuvant therapy.

## 2 METHODS

### 2.1 Data source and collection

Data of patients diagnosed with laryngeal carcinoma (site code C32.0, C32.1, C32.2 C32.3, and C32.8) diagnosed from 2004 to 2015 were collected from the Surveillance, Epidemiology, and End Results (SEER) database, which is an authoritative information source affiliated to the National Cancer Institute (NCI) and is considered to be the golden standard for tumor clinical and pathological information collection worldwide.^13^ The extent of surgery was evaluated using regional nodes positive (1988+) and RX Summ‐Surg Prim Site (1998+). Only patients who received partial or total laryngectomy as their primary therapy and diagnosed with positive histology were included in the patient cohort. Patients were excluded if the following criteria were involved: (a) unknown/obscure follow‐up time; (b) diagnosed autopsy/death certificate only (DCO); (c) not the first tumor; (d) distant metastasis; (e) no lymphadenectomy or ELNs = 0; (f) unknown information on extracted data. Patients with overall survival ≤3 months were also excluded to reduce the potential bias causing by heterogeneity in treatment quality and perioperative care.

Information regarding age at diagnosis, race, gender, primary location, differentiated grade, American Joint Committee on Cancer (AJCC) TNM staging system (6th/7th edition), tumor size, number of metastatic lymph nodes, number of examined lymph nodes, therapy details (surgery record and adjuvant treatment record), and oncological outcomes were collected from the SEER database. In this study, overall survival, which was defined as the period from histological diagnosis date to the date of all‐cause of death or the last follow‐up time, was defined as the endpoint for LC patients.

### 2.2 Statistical analysis

In the baseline characteristics, categorical variables were described as frequency and percentage and continuous variables were represented by the medians. The X‐tile program (Yale University School of Medicine, New Haven, CT, USA) was used to define the optimal cut‐off points for ELNs and MLNR with minimum *P* values for the log‐rank test and the highest specificity and sensitivity. Multivariate cox proportional hazards regression was performed to identify potential prognostic factors. Hazard ratios (HRs) and 95% confidence intervals (CIs) were also calculated. A novel survival‐predicting model was established. The predicting performance of this model was evaluated by the calibration curve and the Harrell's concordance index (C‐index). The Kaplan‐Meier analysis and the log‐rank tests were used to compare the survival differences between subgroups.

All statistical analyses above were performed using SPSS version 22.0 (IBM Corporation, Armonk, NY, USA). R (version 3.5.1; https://www.r%2010project.org) was used to establish and validate the predicting model. All analyses were two‐tailed, a *P*‐value < .05 was considered as statistically significant.

## 3 RESULTS

### 3.1 Clinical characteristics of patients

After meeting the preset criteria, including without exact follow‐up time; autopsy/death certificate only (DCO), a total of 5746 cases receiving partial or total laryngectomy between January 2004 and December 2015 were retrieved from the SEER database. Then patients without lymphadenectomy or without exactly ELNs/LNR (n = 1793), with unknown cause of death and not first tumor (n = 780), unknown information on basic variables (race, differentiation grade, tumor location, TNM stage or tumor size [n = 941]), and patients with overall survival ≤3 months (n = 73) and patients with distant metastases (n = 37) were excluded.

Overall, a total of 2122 patients with resected laryngeal carcinoma (LC) were retrieved from the SEER database. The clinical and pathological characteristics of the patient cohort are summarized in Table 1. Nearly half of the patients (n = 1062) had metastatic lymph nodes. The mean ELNs was 41 (range from 1 to 97), and the mean LNR was 0.066 (range from 0 to 1). Partial laryngectomy was performed in 476 patients and total laryngectomy in 1646 patients. Of 1512 patients received adjuvant therapy, including radiotherapy (n = 816), chemotherapy (n = 36), and chemoradiotherapy (n = 660). The median age at diagnosis was 59 (range from 16 to 92).

**Table 1:** Clinical characteristics of patients with resected laryngeal carcinoma

	Total		Training set		Testing set		*P* value
	2122	%	1485	%	637	%	
Age							
≤50	269	12.7	206	13.9	63	9.9	.001
(50,65)	1181	55.7	789	53.1	392	61.5	
>65	672	31.7	490	33.0	182	28.6	
Race							
White	1621	76.4	1143	77.0	478	75.0	.052
Black	412	19.4	290	19.5	122	19.2	
Other	89	4.2	52	3.5	37	5.8	
Sex							
Female	400	18.9	283	19.1	117	18.4	.762
Male	1722	81.1	1202	80.9	520	81.6	
Year of diagnosis							
≤2010	1189	56.0	841	56.6	348	54.6	.417
>2010	933	44.0	644	43.3	289	45.4	
Primary location							
Supraglottic	1006	47.4	701	47.2	305	47.9	.290
Glottic	810	38.2	556	37.4	254	39.9	
Subglottic	88	4.1	65	4.4	23	3.6	
Other	218	10.3	163	11.0	55	8.6	
Histologic type							
Squamous cell	2048	96.5	1432	96.4	616	96.7	.798
Other	74	3.5	53	3.6	21	3.3	
Grade							
Well	155	7.3	110	7.4	45	7.1	.113
Moderately	1271	59.9	887	59.7	385	60.4	
Poorly	677	31.9	480	32.2	197	30.9	
Undifferentiated	18	0.8	8	0.5	10	1.6	
T classification							
T1	141	6.6	98	6.6	43	6.8	.244
T2	266	12.5	182	12.3	84	13.2	
T3	554	26.1	372	25.1	182	28.6	
T4	1161	54.7	833	56.1	328	51.5	
ELNs							
Median (range)	40	0‐97	39	0‐97	41	0‐97	.167
MLNR							
Median (range)	0.01	0‐1	0.01	0‐1	0.01	0‐1	.106
Treatment							
With adjuvant therapy	610	28.7	443	29.8	167	26.2	.094
Without adjuvant therapy	1512	71.3	1042	70.2	470	73.8	

### 3.2 Identification of the cut‐off points for examined lymph nodes number and metastatic lymph nodes ratio

The X‐tile program was applied to analyze the data of LC patients from the SEER database and to determine the optimal cut‐off values for ELNs and MLNR. The whole patient cohort was randomly divided into a training set containing 1485 patients (70%), and a validating set including 637 patients (30%). The two sets were comparable in the clinical and oncological characteristics (shown in Table 1).

**Figure 1:**
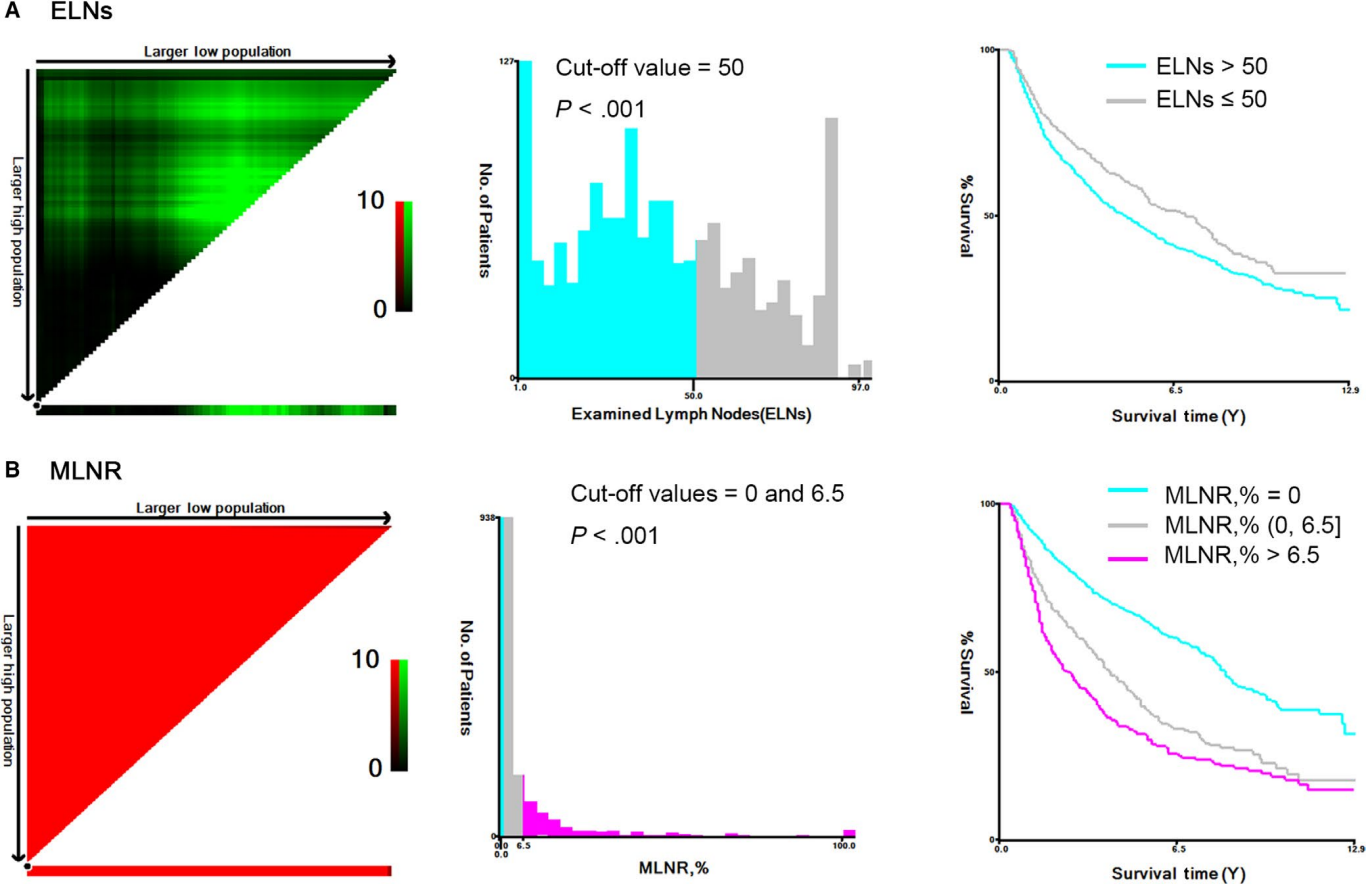
X‐tile analysis determining optimal ELNs and MLNR cut‐off values based on the overall survival. (A) ELNs: 50; (B) MLNR: 0 and 6.5%

For patients in the training set, ELNs and MLNR were enrolled into the X‐tile program for analysis. The results showed that 50 was the optimal cut‐off values for ELNs (*P*‐value < .001, Figure 1A). And the best cut‐off values for MLNR were 0 and 0.065 (*P*‐value < .001, Figure 1B). Univariate and multivariate analysis showed that ELNs (*P* = .006), MLNR (*P* < .001), age (*P* < .001), T stage (*P* < .001) were independent prognostic factors for overall survival in patients with resected laryngeal cancer as listed in Table 2.

**Table 2:** Univariate and multivariate analysis of risk factors for overall survival in patients who underwent laryngectomy and lymphadenectomy

	Univariate analysis		Multivariate analysis	
	Hazard ratio (95% CI)	*P* value	Hazard ratio (95% CI)	*P* value
Age				
≤50	(Ref)		(Ref)	
(50,65)	1.456 (1.155‐1.836)	.001	1.292 (1.023‐1.632)	.031
>65	2.085 (1.643‐2.645)	.000	1.877 (1.474‐2.390)	.000
Race				
White	(Ref)			
Black	1.151 (0.970‐1.366)	.107		
Other	1.079 (0.733‐1.587)	.700		
Sex				
Female	(Ref)			
Male	1.161 (0.968‐1.391)	.107		
Primary location				
Supraglottic	(Ref)			
Glottic	0.912 (0.782‐1.063)	.238		
Subglottic	1.059 (0.752‐1.492)	.742		
Other	1.207 (0.963‐1.512)	.103		
Histologic type				
Squamous cell	(Ref)			
Other	1.374 (0.974‐1.938)	.070		
Grade				
Well	(Ref)		(Ref)	
Moderately	1.497 (1.087‐2.060)	.013	1.368 (0.991‐1.887)	.057
Poorly	1.853 (1.335‐2.572)	.000	1.432 (1.025‐1.999)	.035
Undifferentiated	2.728 (1.158‐6.428)	.022	1.884 (0.794‐4.469)	.151
T classification				
T1	(Ref)		(Ref)	
T2	1.716 (1.148‐2.565)	.008	1.625 (1.085‐2.434)	.019
T3	2.085 (1.442‐3.016)	.000	1.894 (1.308‐2.743)	.001
T4	2.489 (1.749‐3.544)	.000	2.339 (1.641‐3.334)	.000
ELNs				
≤50	(Ref)		(Ref)	
>50	0.769 (0.660‐0.896)	.000	0.794 (0.674‐0.937)	.006
MLNR				
0	(Ref)	.000	(Ref)	
(0, 0.065)	1.858 (1.605‐2.150)	.000	1.985 (1.654‐2.382)	.000
>0.065	2.523 (2.196‐2.900)	.000	2.307 (1.946‐2.735)	.000

### 3.3 Generation of a new survival‐predicting model

**Figure 2:**
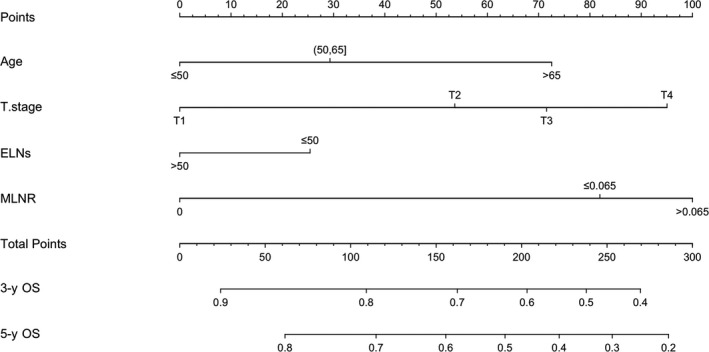
Overall survival‐predicting model for LC patients who had undergone laryngectomy and lymphadenectomy

Based on the result of multivariate analysis, prognostic factors, including ELNs, MLNR, age, T stage were utilized to construct a new survival predicting model (Figure 2). Each independent parameter was allotted a score, and the total risk point can be calculated by adding all the variable scores. Finally, the probability of death in each patient can be directedly read by drawing a plummet line.

**Figure 3:**
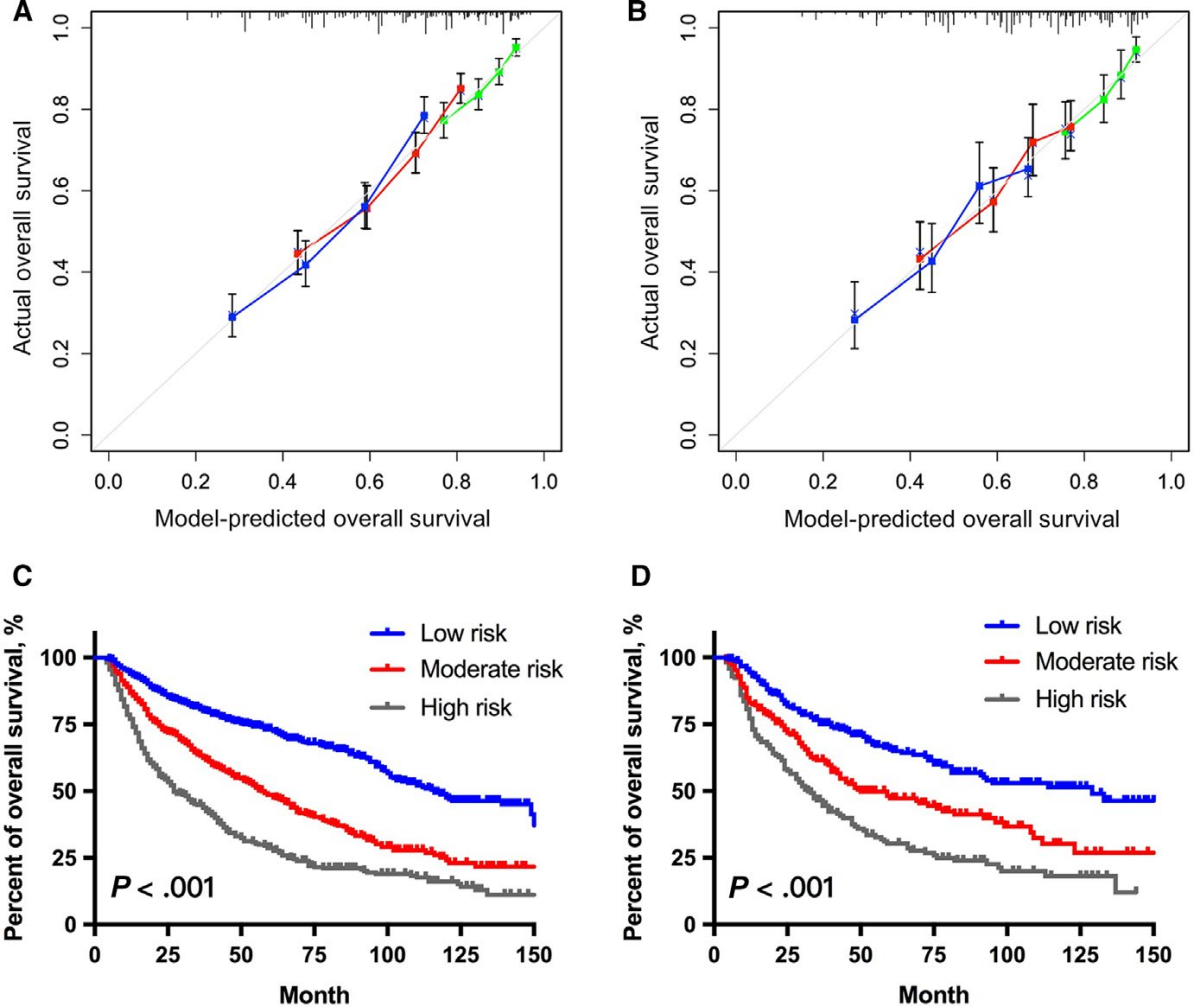
The calibration curve of the model for predicting overall survival in the training set (A) and in the testing set (B). Model‐predicted OS is plotted on the x‐axis, and actual on the y‐axis. Comparison of Kaplan‐Meier curves between subgroups in the training set (C) and in the testing set (D)

The predicting performance of the model was assessed using the C‐index and calibration curve, and internally validated in the training set. In the training set, a C‐index of 0.683 (95% CI: 0.663‐0.703) was observed for the model, which was higher than that of the AJCC TNM stage system (7th) (0.641, 95% CI: 0.621‐0.661). For the testing set, the survival‐predicting performance of our model (0.662, 95% CI: 0.631‐0.693) was still superior to that of the AJCC TNM stage system (7th) (0.641, 95% CI: 0.621‐0.661). The calibration curves showed that the predicted OS by our model matched well with clinically observed OS in both training set and testing set (Figure 3A,B).

In addition to predicting probability of OS, this model was also used to classify patients into three subgroups with the incremental risk of death based on their total scores. The stratification strategy was as follows: (a) high‐risk group: >210 (n = 621), (b) moderate‐risk group: (150, 210] (n = 696), and 3) low‐risk group: ≤150 (n = 805). Significant survival differences were observed between subgroups in the training set and validating set (Figure 3C,D).

### 3.4 Clinical value of the novel classification system

**Figure 4:**
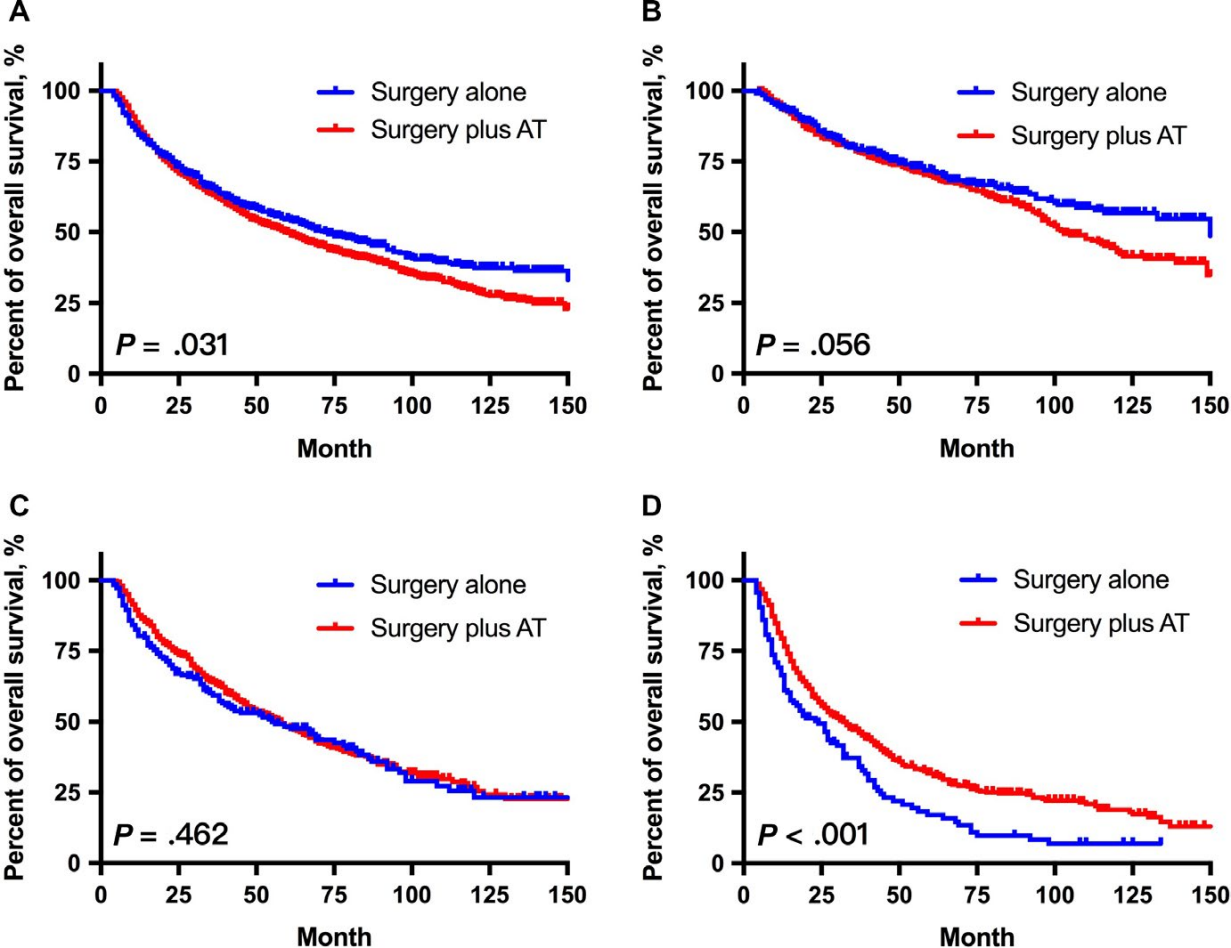
The Kaplan‐Meier curves of OS for patients after laryngectomy and lymphadenectomy. The prognostic value of adjuvant therapy (AT) in total patient cohort (n = 2122, A), low‐risk group (n = 805, B), moderate‐risk group (n = 696, C), and high‐risk group (n = 621, D)

Overall, the 3‐year and 5‐year OS of patients with resected laryngeal carcinoma were 63.8% and 51.1%, respectively. Kaplan‐Meier analysis and the log‐rank test showed that patients receiving adjuvant therapy had even worse survival outcomes than those receiving surgery alone (*P* = .031, Figure 4A). It was imperative to select suitable patients for adjuvant therapy in case of the occurrence of excessive medical treatment.

Next, we further compared the survival outcomes between patients with and without adjuvant treatment in each subgroup based on our novel survival‐predicting model. Notably, only patients in high‐risk group could benefit from adjuvant treatment (*P* < .001, Figure 4D), whereas patients in other groups did not (Figure 4B,C). In the high‐risk group, the 3‐year survival of patients receiving adjuvant therapy was 47.2%, which was higher than that of patients receiving surgery alone (37.3%). Notably, for patients in low‐risk group, the long‐term survival outcomes of those receiving adjuvant therapy (10‐year OS: 43.0%) were even worse than those receiving surgery alone (10‐year OS: 56.8%). These findings supported that the stratification strategy could help to guide adjuvant treatment for patients with resected laryngeal cancer.

## 4 DISCUSSION

In this study, the data of patients with resected laryngeal carcinoma were retrieved from the SEER database to investigate the prognostic value of lymph node status. We found that lymphatic metastasis was relatively common among patients receiving both laryngectomy and lymphadenectomy, and there were more than 50% of patients who had at least one positive lymph node. Under this background, how to accurately evaluate the prognostic significance of lymph node status has become an urgent problem to be solved.

Lymph node status is relatively important for laryngeal cancer, including the number of examined lymph nodes and the ratio of metastatic lymph nodes. Notably, the exact number of pathologically confirmed lymph nodes has been incorporated into the AJCC TNM stage system.^14^ However, accurate quantification of metastatic lymph nodes largely depends on a sufficient number of ELNs. When the number of harvested lymph nodes is insufficient, potential positive lymph nodes may be ignored, which may result in the overestimation of oncological outcomes and affect the decision for subsequent treatment. The finding of our study suggested that 50 was considered as the optimal cut‐off value of ELNs for patients with resected laryngeal cancer. The 10‐year cumulative incidence of all‐cause of death in patients with ELNs> 50 was 70.0%, whereas in the meantime a 4.8% risk increment of that in patients with ELNs ≤50. This finding supported that systematic lymphadenectomy (ELNs> 50) could help improve the oncological outcomes of patients with laryngeal carcinoma.

Besides, the ratio of metastatic lymph nodes was also found to be an important prognostic factor for laryngeal cancer and many other cancers.^8^, ^15^ Prabhu et al indicted higher MLNR (>0.20) were associated with a higher risk of locoregional recurrence and death in patients with head and neck carcinoma after primary surgical resection.^16^ Ryu et al also identified MLNR as an independent prognostic factor of cancer‐specific mortality in pN+ patients with laryngeal squamous cell cancer after laryngectomy.^17^ Thus, in this study, we jointly use ELNs, MLNR, and other clinicopathological features (age and T stage) to construct a novel survival‐predicting model, which was superior in predicting oncological outcomes of patients with resected laryngeal cancer. Notably, we further proposed a novel patient classification strategy based on the model. Patients were stratified into three subgroups with different risk of death. Kaplan‐Meier analysis and log‐rank tests showed significant survival differences between subgroups.

The purpose of adjuvant therapy should be the elimination of subclinical lesions. However, the adverse effects it may induce limit its application in most patients. The implementation of adjuvant therapy is still in the dilemma of the potential risk of undertreatment and excessive medical treatment. Agencies hold various opinions upon the role of adjuvant therapy for patients with resected laryngeal carcinoma. For instance, the American College of Radiology (ACR) recommends adjuvant treatment for advanced‐stage laryngeal cancer,^18^ whereas the National Comprehensive Cancer Network (NCCN) considers adjuvant therapy as an optimal option only for patients with certain adverse features, such as extracapsular nodal spread and positive margin.^19^ In this study, the survival outcomes of patients receiving surgery alone were even better than those receiving adjuvant therapy after initial surgical resection. From our perspective, this might be caused by two reasons, that is patients who were suggested to receive adjuvant therapy had more aggressive tumor progression in most cases and the adverse effects of adjuvant treatment might impair the prognosis of patients who did not candidate for it. How to select candidates for adjuvant therapy remains controversial.

Theoretically speaking, the presence of subclinical foci is determined by tumor behavior and the extent of surgery. Tumor behavior can be reflected by MLNR and T stage to some extent. ELNs reflects the adequacy of resection clearance in a way. So, in this study, we jointly used ELNs, MLNR, T stage, and age to distinguish patients with potential subclinical lesions and predict the role of adjuvant therapy in different patients. Patients with resected laryngeal carcinoma were classified into three groups with increasing risk of death. Then we investigate the effect of adjuvant therapy in each group. Notably, only patients in the high‐risk group were found to benefit from adjuvant treatment significantly (*P* < .001), so we strongly recommend patients in the high‐risk group as candidates for adjuvant therapy. While for patients in the moderate‐risk group, the survival difference was not statistically significant, so surgery alone and regular examination were recommended, and adjuvant treatment was only used for those with other adverse characteristics, such as positive margin and extracapsular extension. As for patients in the low‐risk group, adjuvant therapy was not beneficial and even impaired the long‐term survival of patients to some extent, so surgery alone was sufficient for those patients.

Our study still had several limitations that should be highlighted, including its retrospective nature and the potential selection biases. Furthermore, patients included in our study were treated in different medical centers. Therefore, the quality of operations and adjuvant treatments and the methods employed by pathologists for diagnosing metastatic lymph nodes were not unified, which might influence the results of our study to some extent. In addition, the data of our study were retrieved from the SEER database, which lacked the information of extranodal extension, so we failed to restage patients according to the 8th AJCC stage system and compare the predicting performance of our new model with that of the 8th AJCC staging classification. Further prospective studies are required to testify whether our new model is superior to the conventional AJCC 8th TNM staging system in stratifying patients and in determining the value of adjuvant therapy after the initial surgery. Finally, although univariate analysis showed that primary location was not an independent prognostic factor for patients with resected laryngeal cancer in our study, other studies have indicated that each location corresponds to a different pattern of regional spread as well as different overall survival.^2^, ^4^ If all sublocations are included together, there may exist over or infra estimation of the real overall survival. Further prospective studies are also required to testify its real estimation capacity in each sublocation, respectively. Nevertheless, our study was valuable and was the first to jointly use lymph nodes statue and other clinicopathological characteristics to evaluate the prognosis of patients after laryngectomy and lymphadenectomy and predict the prognostic value of adjuvant therapy.

## 5 CONCLUSION

In this large population‐based study, we proposed a novel model to predict the survival outcomes for patients with resected laryngeal carcinoma and assist in the decision making of adjuvant treatment. Further studies to validate and improve this model were warranted.

## CONFLICT OF INTEREST

The authors report no conflicts of interest. The authors are responsible for the content and writing of the paper.

## Data Availability

The data that support the findings of this study are available from the corresponding author upon reasonable request.
